# Response to Integration of the Comprehensive Physical Examination and Diagnostic Tools in Clinical Cardiology

**DOI:** 10.1002/kjm2.70075

**Published:** 2025-07-13

**Authors:** Yu‐Shien Kao, Yi‐Hsun Chen, I‐Chen Wu

**Affiliations:** ^1^ Kaohsiung Medical University Chung‐Ho Memorial Hospital, Kaohsiung Medical University Kaohsiung Taiwan; ^2^ Kaohsiung Medical University Kaohsiung Taiwan

Dear Editor,

We sincerely thank the readers for their thoughtful response to our recent case report, “Kussmaul's sign provoked by intracardiac metastasis of endometrial neuroendocrine tumor” [1]. Their insightful comments on the historical and physiological interpretation of jugular venous findings provide a valuable opportunity to revisit and clarify the nuances surrounding Kussmaul's sign and related physical examination findings.

We fully agree that terminological precision is essential in medical communication and education. The original context in which Adolf Kussmaul described a paradoxical inspiratory rise in jugular venous pressure—namely, mediastino‐pericarditis with adhesion—should indeed be recognized as foundational. However, as the understanding of pathophysiological mechanisms has expanded, the term “Kussmaul's sign” has also evolved in clinical practice to describe similar hemodynamic patterns resulting from impaired right ventricular compliance, even in the absence of pericardial disease [2, 3]. This broadened usage, though potentially prone to semantic drift, reflects real‐world application in bedside diagnostics and was used in this sense in our report.

Regarding the specific case, we acknowledge the absence of detailed jugular venous waveforms or ECG tracings in the original report. As noted by the readers, such information would certainly enhance interpretive clarity. However, the accompanying video clip (submitted as Supporting Information) was intended to allow readers to directly observe the physical sign in question. As the readers correctly noted, the inspiratory distention of the jugular vein suggests elevated right‐sided filling pressure, likely in the setting of preserved atrial contraction—an observation we fully support.

While we agree that further rhythm analysis (e.g., ECG documentation) would help distinguish between sinus rhythm, atrial fibrillation, or other arrhythmic causes of abnormal venous pulsation, we also emphasize the clinical context. The patient's intracardiac mass was directly impinging on right heart inflow, and the visible pulsation patterns were consistent with mechanical impedance to right ventricular diastolic filling. Given the advanced malignancy and the urgent need for tumor control, additional invasive or electrophysiological studies were deferred.

We are grateful for the readers' historical overview of jugular venous physiology and its clinical implications. Their discussion reinforces the enduring value of physical examination in cardiovascular assessment, a sentiment we share and hoped to emphasize through our case.

Once again, we thank the authors for their enriching perspective, which meaningfully advances the discussion surrounding this classical but often misunderstood clinical sign.

## Conflicts of Interest

The authors declare no conflicts of interest.

## Supporting information


**Video S1.** Unusual pulsatile activity in the neck. As the tumor progressed, she developed symptoms including dyspnea and her jugular venous pressure paradoxically increased during inspiration, consistent with Kussmaul’s sign.

## Data Availability

The data that support the findings of this study are available on request from the corresponding author. The data are not publicly available due to privacy or ethical restrictions.
