# Correction to “Response to Integration of the Comprehensive Physical Examination and Diagnostic Tools in Clinical Cardiology”

**DOI:** 10.1002/kjm2.70164

**Published:** 2025-12-26

Y.‐S. Kao, Y.‐H. Chen, and I.‐C. Wu, “Response to Integration of the Comprehensive Physical Examination and Diagnostic Tools in Clinical Cardiology,” *The Kaohsiung Journal of Medical Sciences* 41, no. 11 (2025): e70075, https://doi.org/10.1002/kjm2.70075.

Yu‐Shien Kao^1^ | Yi‐Hsun Chen^1,2^ | I‐Chen Wu^1,2^

^1^Kaohsiung Medical University Hospital, Kaohsiung Medical University, Kaohsiung, Taiwan

^2^Kaohsiung Medical University, Kaohsiung, Taiwan

The affiliations were listed incorrectly and should be revised to:

^1^Department of Internal Medicine, Kaohsiung Medical University Hospital, Kaohsiung, Taiwan

^2^College of Medicine, Kaohsiung Medical University, Kaohsiung, Taiwan

We apologize for this error.

